# Cell Surface Expression and PET Imaging Establish DLL3 as a Clinically Actionable Target in Neuroblastoma

**DOI:** 10.2967/jnumed.126.272770

**Published:** 2026-11

**Authors:** Salvatore M. Aspromonte, Tamar Y. Feinberg, Somali Gavane, Candace Parker, Ali Cihan, Daoqi You, Salomon A. Tendler, Jaime Alvarez-Perez, Samantha Brosius, Armaan Siddiquee, Dylan Domenico, Glorymar Ibañez, Emily Stockfisch, James M. Bean, Anne M. Cochrane, Umesh Bhanot, Satish K. Tickoo, Achim Jungbluth, Denise Frosina, Paola Angelini, Neerav Shukla, Serge K. Lyashchenko, John T. Poirier, Heiko Schoder, Charles M. Rudin, Jason S. Lewis, Kevin J. Curran, Anthony Daniyan, Mark P. Dunphy, Julia L. Glade Bender, Shakeel Modak, Filemon S. Dela Cruz, Andrew L. Kung

**Affiliations:** 1 Department of Pediatrics, Memorial Sloan Kettering Cancer Center, New York, New York;; 2 Department of Radiology, Molecular Imaging and Therapy Service, Memorial Sloan Kettering Cancer Center, New York, New York;; 3 Molecular Pharmacology Program, Memorial Sloan Kettering Cancer Center, New York, New York;; 4 Department of Medicine, Memorial Sloan Kettering Cancer Center, New York, New York;; 5 Department of Epidemiology and Biostatistics, Memorial Sloan Kettering Cancer Center, New York, New York;; 6 Immunology Program, Memorial Sloan Kettering Cancer Center, New York, New York;; 7 Department of Pathology and Laboratory Medicine, Memorial Sloan Kettering Cancer Center, New York, New York;; 8 Radiochemistry and Molecular Imaging Probes Core, Memorial Sloan Kettering Cancer Center, New York, New York; and; 9 Department of Medicine, NYU Grossman School of Medicine, New York, New York

**Keywords:** DLL3, neuroblastoma, PET imaging

## Abstract

In adult neuroendocrine malignancies, including small cell lung cancer, Delta-like ligand 3 (DLL3) is a validated therapeutic target. DLL3 is expressed in neuroblastoma, a pediatric cancer of neuroendocrine origin, but subcellular localization is incompletely defined, limiting clinical translation of DLL3-targeted therapies for neuroblastoma. **Methods:** DLL3 expression and localization were evaluated in human neuroblastoma tumors and preclinical models using RNA sequencing, immunohistochemistry, and flow cytometry. To assess in vivo tumor accessibility, DLL3-targeted PET imaging with [^89^Zr]Zr-deferoxamine-SC16.56 was performed in patient-derived xenograft (PDX) models and in 4 patients with neuroblastoma. **Results:** DLL3 was broadly expressed across neuroblastoma tumor samples and preclinical models. Although immunohistochemistry demonstrated variable protein expression, flow cytometry revealed near-universal DLL3 cell surface localization across clinical samples and PDX models, including tumors with low expression by immunohistochemistry. DLL3-targeted PET imaging demonstrated tumor-specific uptake in PDX models and in 4 patients with relapsed neuroblastoma, confirming in vivo accessibility of DLL3. **Conclusion:** DLL3 overexpression in neuroblastoma is associated with targetable cell surface localization, supporting DLL3 as a clinically actionable target for imaging and therapeutic strategies.

## Visual Abstract

**Figure:**
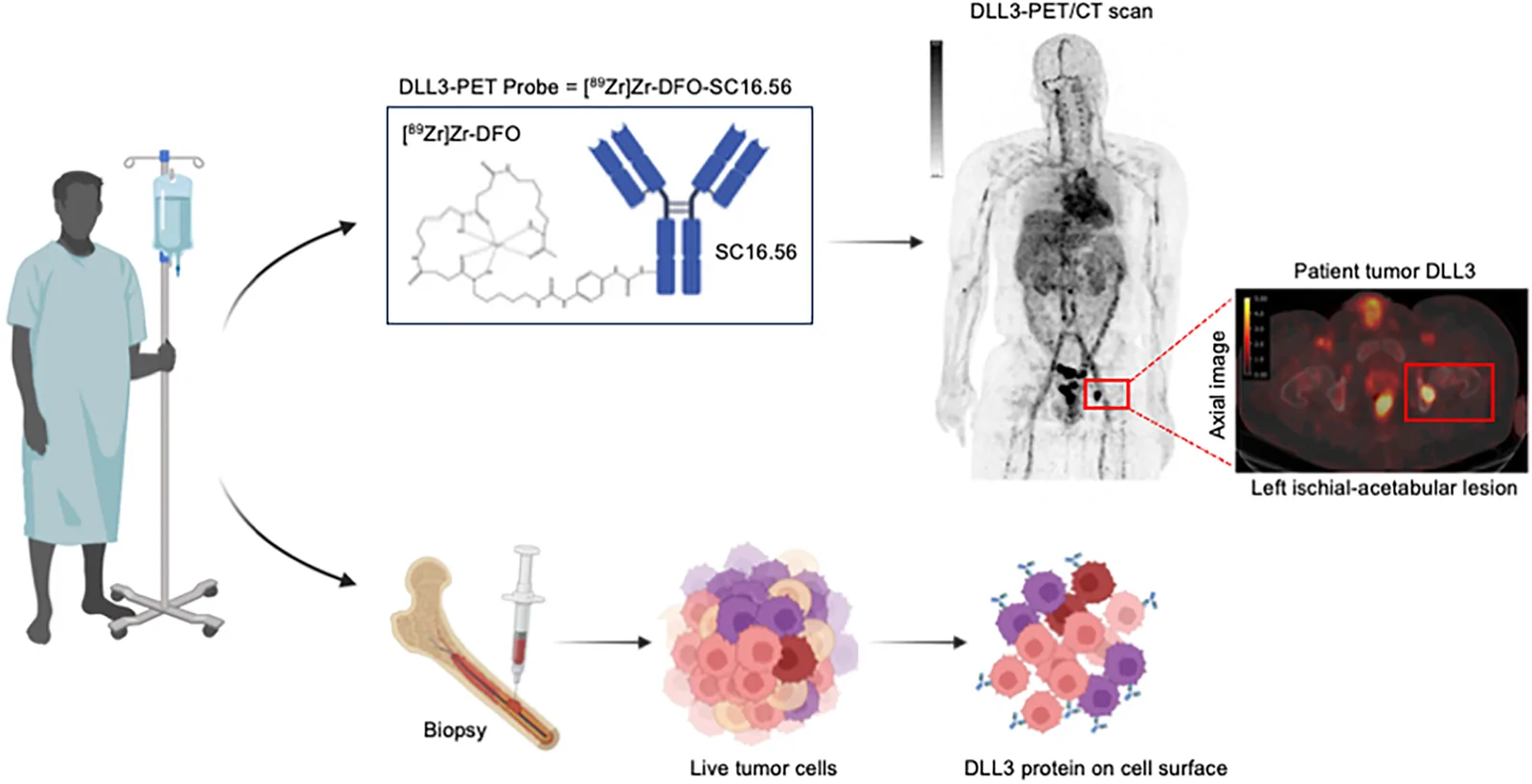

An inhibitory ligand of the canonical Notch pathway, Delta-like ligand 3 (DLL3), disrupts cell differentiation and promotes tumorigenesis when aberrantly overexpressed (1). DLL3 is a validated therapeutic target in adult neuroendocrine cancers because of its preferential expression on malignant cells and minimal expression in normal tissues (1–4). Multiple DLL3-targeting therapeutics have been evaluated in adult neuroendocrine malignancies, including the DLL3-targeting bispecific antibody, tarlatamab, which achieved Food and Drug Administration approval for treatment of patients with small cell lung cancer (5,6). The tumor specificity of DLL3 has been corroborated by DLL3 PET imaging using [^89^Zr]Zr-deferoxamine-SC16.56, which demonstrated tumor uptake in 83% of patients with neuroendocrine lung and prostate malignancies (7) and in 83% of patients with gastroenteropancreatic neuroendocrine neoplasms with high tumor-to-background ratios in all cohorts (8).

Neuroblastoma, the most common extracranial solid tumor in children (9), arises from the sympathetic nervous system and is neuroendocrine in origin. Neuroblastoma has been shown to express DLL3 messenger RNA at levels comparable to those of small cell lung cancer, and preclinical studies evaluating a DLL3 antibody–drug conjugate in neuroblastoma demonstrated antitumor activity in select models (4). However, concerns about heterogeneous DLL3 expression in preclinical models and uncertainty with regard to cell surface DLL3 protein expression in human tumors have limited further evaluation of DLL3 targeting in neuroblastoma (4).

Given the unmet need for more effective treatments for patients with high-risk neuroblastoma (9,10) and existing questions about the subcellular location of DLL3 in tumor cells (11–15), we undertook a systematic assessment of DLL3 expression and tumor cell surface localization in human neuroblastoma samples and preclinical models. We further assessed the in vivo accessibility of DLL3 as a tumor-specific target in preclinical models and in patients with neuroblastoma.

## MATERIALS AND METHODS

### Patient Consent for Sequencing and Research

Patients undergoing clinically indicated procedures were identified and approached for informed consent for participation in Memorial Sloan Kettering Cancer Center (MSK) clinical sequencing (MSK institutional review board number 12-245, NCT01775072) and biospecimen research studies (institutional review board number 06-107).

### Patient Tumor Processing

Surgically resected specimens and biopsy samples obtained for clinical diagnostic purposes were allocated for patient-derived xenograft (PDX) generation and tumor biobanking when sufficient tissue remained after clinical work-up. Molecular profiling of formalin-fixed and paraffin-embedded tumor tissue was performed using next-generation targeted sequencing (16).

### RNA Sequencing

RNA sequencing data from clinically annotated tumor specimens sequenced at MSK were accessed through the Isabl database (Isabl Inc.), as previously described (17,18). Normalized gene expression values were extracted directly from the Isabl database for analyses (17).

### PDX Tumor Microarray Construction

PDX tissue microarrays were constructed using formalin-fixed and paraffin-embedded PDX tissues, as previously described (19).

### Immunohistochemistry

DLL3 immunohistochemistry was performed using clone SP347 on the BenchMark Ultra platform (Ventana Medical Systems). Staining was scored by pathologists using a histochemical score (0–300), with positivity defined as a score greater than 10.

### Flow Cytometry

DLL3 recombinant human monoclonal primary antibody (clone 7B7, catalog number MA5-43156, research resource identifier AB_2912292; Thermo Fisher Scientific) was conjugated to phycoerythrin–cyanine 7 using a Lightning-Link antibody labeling kit (product number ab102903; Abcam). DLL3 expression was analyzed on viable CD45-negative, CD56-positive tumor cells using phycoerythrin-conjugated antihuman CD45 (clone H130, catalog number 304008, research resource identifier AB_314396; BioLegend), allophycocyanin-conjugated antihuman CD56/NCAM-1 (clone B159, catalog number 555518, research resource identifier AB_398601; BD Biosciences), phycoerythrin–cyanine 7–conjugated DLL3 (catalog number MA5-43156; Invitrogen), or phycoerythrin–cyanine 7–conjugated human IgG isotype control (catalog number 31154, research resource identifier AB_243591; Thermo Fisher Scientific).

### Synthesis of [^89^Zr]Zr-Deferoxamine-SC16.56 and Dose Preparation

SC16.56, a humanized anti-DLL3 monoclonal antibody (Stemcentrx Inc.), was conjugated with deferoxamine and radiolabeled with ^89^Zr to generate [^89^Zr]Zr-deferoxamine-SC16.56. Radiotracer production and DLL3 PET probe preparation were performed as previously described (7,8). [^89^Zr]Zr-deferoxamine-SC16.56 underwent quality control testing before patient administration.

### DLL3 PET/CT Imaging of PDX Mice

Animal studies with radiolabeled [^89^Zr]Zr-deferoxamine-SC16.56 were performed in accordance with MSK Institutional Animal Care and Use Committee–approved protocol number 08-07-013. PDX mice underwent serial PET/CT imaging at 24, 72, and 120 h after tail vein injection of 3.7 MBq (100 µCi, 20 µg) of [^89^Zr]Zr-deferoxamine-SC16.56, coadministered with 850 µg of unlabeled nonspecific human IgG1 to minimize nonspecific binding. PET acquisitions were performed to a target of 40,000 counts per mouse. Images were reconstructed and quantified as percentage injected dose per gram of tissue (%ID/g). Biodistribution analyses were performed after 120 h.

### Human DLL3/PET Studies

Four patients with neuroblastoma were enrolled in an ongoing single-center phase 1/2 immuno-PET study (Immuno-PET Imaging of Neuroendocrine Tumors Using [^89^Zr]Zr-Deferoxamine-SC16.56, a DLL3-Targeting Monoclonal Antibody, institutional review board number 19-292), and all subjects gave written informed consent. All patients met the study inclusion criteria, including detectable disease on [^123^I]MIBG scintigraphy with SPECT/CT. On day 0, [^89^Zr]Zr-deferoxamine-SC16.56 (74 MBq, 2 mCi; total antibody mass, 2.5 mg) was administered intravenously over 10 min. Images were obtained 3–5 d postinjection.

### Statistical Methods

The association between DLL3 transcript abundance (in transcripts per million [TPM]) and protein expression (histochemical score) was assessed using the Spearman rank correlation coefficient. Significance was defined as a *P* value of less than 0.05.

## RESULTS

### DLL3 Expression in Patient Neuroblastoma Tumors

To assess the expression of *DLL3* messenger RNA, we analyzed tumor RNA sequencing data across a clinical cohort of 540 patients with pediatric solid tumors. *DLL3* transcripts were detected across multiple pediatric tumor types, including neuroblastoma, central nervous system tumors, and various sarcomas. Among non–central nervous system tumors, neuroblastoma (*n* = 109) had the highest *DLL3* transcript levels (range, 0–19.7 TPM; median, 0.38 TPM), whereas most other pediatric non–central nervous system solid tumors had negligible *DLL3* expression (*n* = 325; median, 0.04 TPM) (Fig. 1A). Central nervous system tumors (*n* = 39) had *DLL3* transcript levels similar to those of neuroblastoma (range, 0–11.7 TPM; median, 0.18 TPM). Assessment of RNA sequencing data from 188 PDX pediatric tumor samples confirmed consistent *DLL3* expression in neuroblastoma (*n* = 25) (Fig. 1B).

**FIGURE 1.**
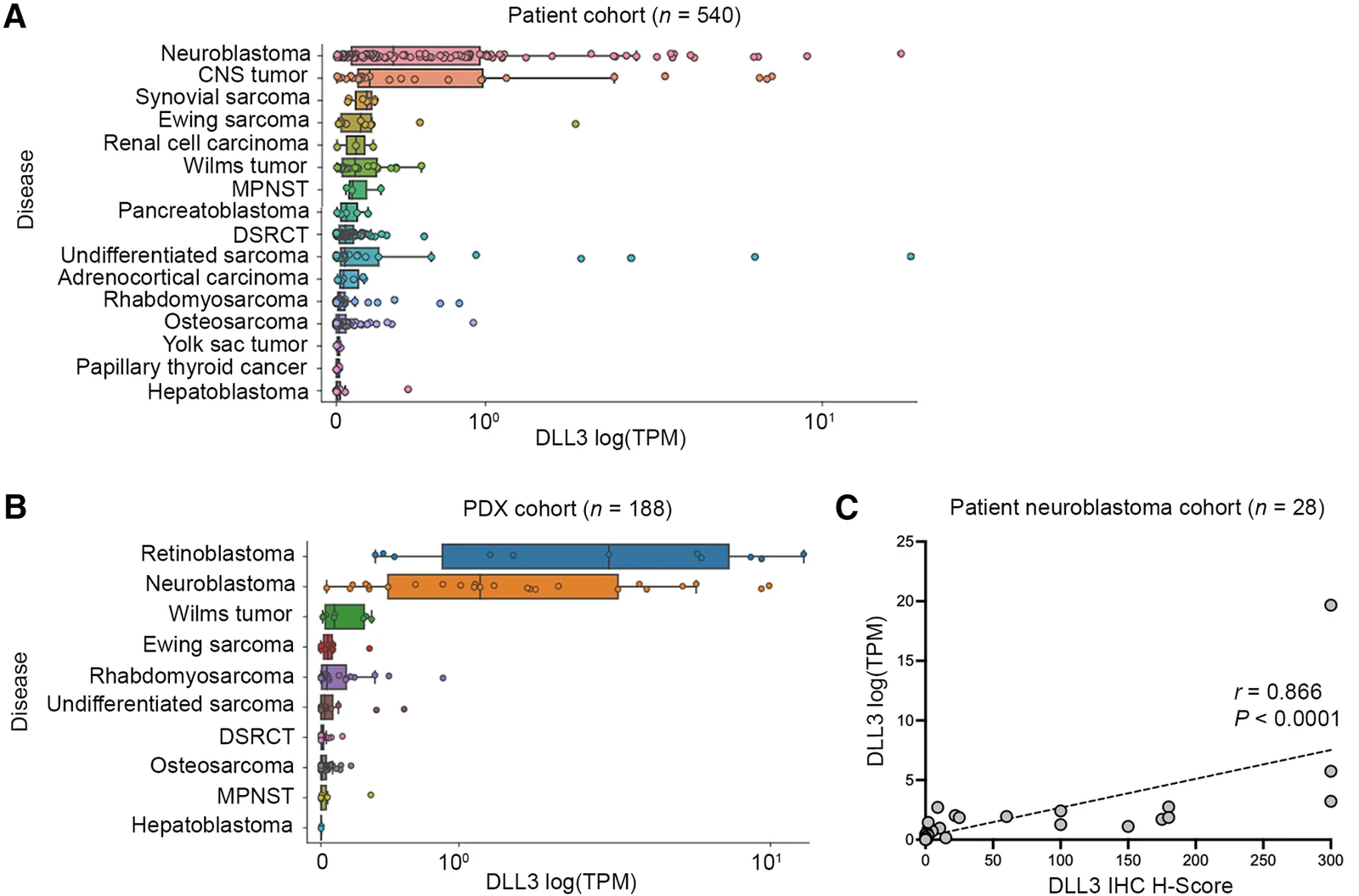
DLL3 transcript levels in tumors of patients with neuroblastoma. (A) Box plots with individual data points showing DLL3 transcript levels (TPM) across pediatric tumor subtypes (*n* = 540). (B) DLL3 transcript levels (TPM) across 188 PDX models by disease subtype. (C) Correlation between DLL3 transcript levels determined by bulk RNA sequencing and DLL3 histochemical scores in 28 patient tumors. CNS = central nervous system; DSRCT = desmoplastic small round cell tumor; H-score = histochemical score; IHC = immunohistochemistry; MPNST = malignant peripheral nerve sheath tumor.

Given that messenger RNA expression does not always correlate with protein abundance, we used immunohistochemistry to evaluate DLL3 protein expression in 28 unique neuroblastoma tumor samples. DLL3 protein expression (histochemical score, ≥10) was detected in 57% of cases (16/28), with histochemical scores ranging from 0 to 300 (median, 100). Comparison of patient-specific RNA sequencing and immunohistochemistry protein expression data revealed a significant correlation across the cohort (Spearman *r* = 0.866, *P* < 0.0001) (Fig. 1C).

### Cell Surface Localization of DLL3 in Patient Neuroblastoma Tumors

To assess cell surface localization, we used flow cytometry to assess DLL3 across neuroblastoma cell lines and patient samples (Supplemental Table 1 [supplemental materials are available at http://jnm.snmjournals.org]), compared with protein abundance assessed by immunohistochemistry (20). Neuroblastoma cell line pellets demonstrated variable DLL3 protein expression by immunohistochemistry, ranging from prominent membranous and cytoplasmic staining (cell lines SK-N-DZ, SK-N-FI, and CHP-212) to negligible staining (cell lines SK-N-BE and SK-N-SH) (Supplemental Fig. 1A). In contrast, flow cytometry identified DLL3-positive tumor cell populations not only in immunohistochemistry-positive cell lines (SK-N-DZ, 10.6%; SK-N-FI, 47.6%; CHP-212, 8.9%) but also in cell lines with low DLL3 or DLL3 undetectable by immunohistochemistry (SK-N-BE, 15.8%; SK-N-SH, 4.6%) (Supplemental Fig. 1B). KELLY cells had no appreciable cell surface DLL3 despite a positive immunohistochemistry signal (histochemical score, 60), and HEK-293T negative control cells lacked DLL3 detectable by either immunohistochemistry or flow cytometry (Supplemental Fig. 1B). The abundance of DLL3-positive tumor cells and mean fluorescence intensity in neuroblastoma cell lines SK-N-DZ and SK-N-SH were comparable to those observed in the small cell lung cancer cell lines H69, H82, and SHP-77 (Supplemental Fig. 2A).

We next assessed DLL3 expression in patient tumors using immunohistochemistry (Fig. 2A). Histochemical scores were determined by a pathologist and ranged from 0 to 175 (Fig. 2A). Flow cytometry was used to determine cell surface localization of DLL3 in dissociated viable patient samples. DLL3 surface expression was detected in all tumors, including those with a histochemical score of 0, with 2%–75% of tumor cells staining positive for DLL3 (Fig. 2B). The 2 tumors with the highest histochemical scores (BB-208752 and BB-206186) had the lowest percentage of surface DLL3-positive cells by flow cytometry. These data indicate that DLL3 is detectable on the cell surface in most neuroblastoma cell lines and patient samples, including models with low DLL3 or DLL3 undetectable by immunohistochemistry.

**FIGURE 2.**
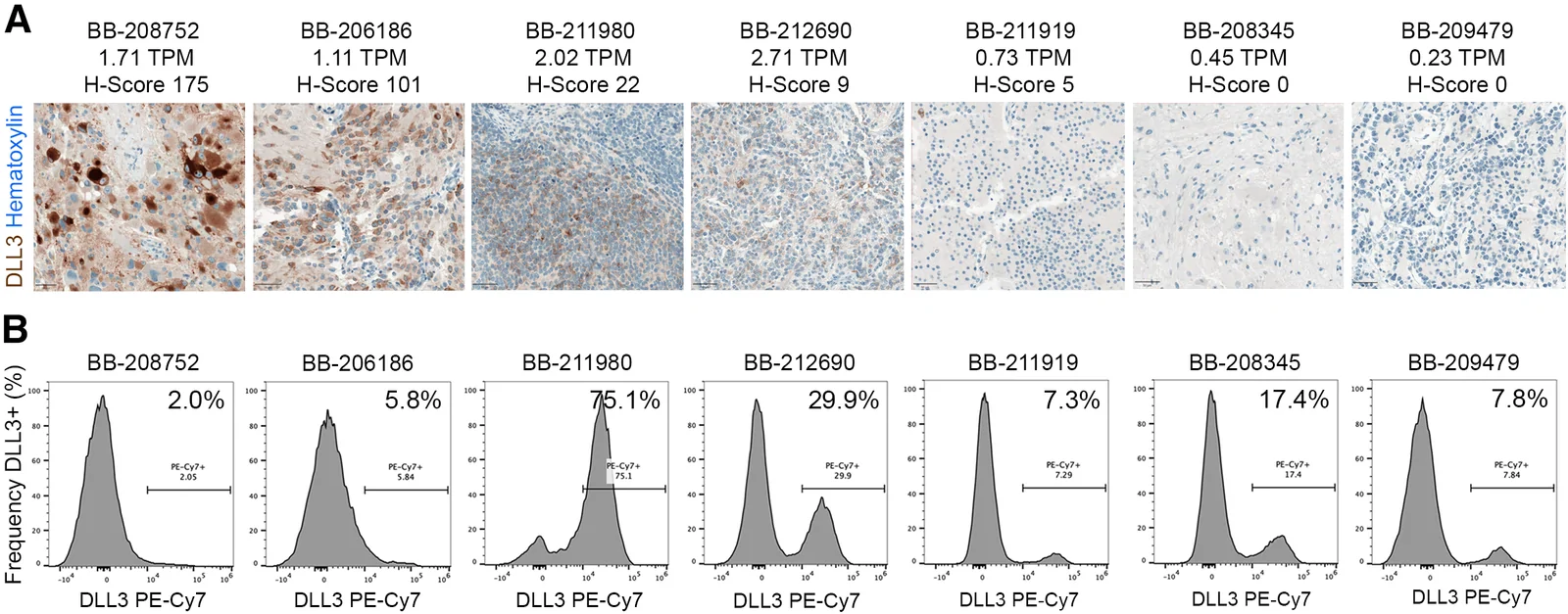
DLL3 expression and cell surface localization in tumors of patients with neuroblastoma. (A) DLL3 IHC images from patient tumors with corresponding DLL3 transcript levels and H-scores (scale bar, 50 µm). (B) Flow cytometric analysis of same tumors stained with anti-DLL3 antibody. Histograms show DLL3-positive cells within CD45-negative, CD56-positive population using modal normalization. H-score = histochemical score; IHC = immunohistochemistry; PE-Cy7 = phycoerythrin–cyanine 7.

### In Vivo Accessibility of DLL3 in Neuroblastoma PDX Models

To determine whether DLL3 was targetable in vivo, we evaluated DLL3 protein expression across our portfolio of pediatric cancer PDX models. DLL3 protein expression was assessed by immunohistochemistry across a pan-histology PDX tissue microarray (*n* = 244, including 30 neuroblastoma cases). Consistent with messenger RNA expression levels in patient samples (Fig. 1B), neuroblastoma PDX tumors were among the highest expressors of DLL3 across pediatric solid tumor types (Fig. 3A), and DLL3 protein levels correlated with RNA expression (Spearman *r* = 0.519, *P* = 0.008) (Fig. 3B). By immunohistochemistry, DLL3 was detected in 59% (17/29) of neuroblastoma PDX tumors, with histochemical scores ranging from 0 to 180 (median, 20). We next assessed surface localization of DLL3 using flow cytometry on 5 neuroblastoma PDX models (all MYCN-amplified). DLL3 was detected on tumor cells across all PDX models, even those with histochemical scores of less than 10 (Fig. 3C), with DLL3-positive tumor cells comprising 25.8%–88.5% of the tumor population (Fig. 3D).

**FIGURE 3.**
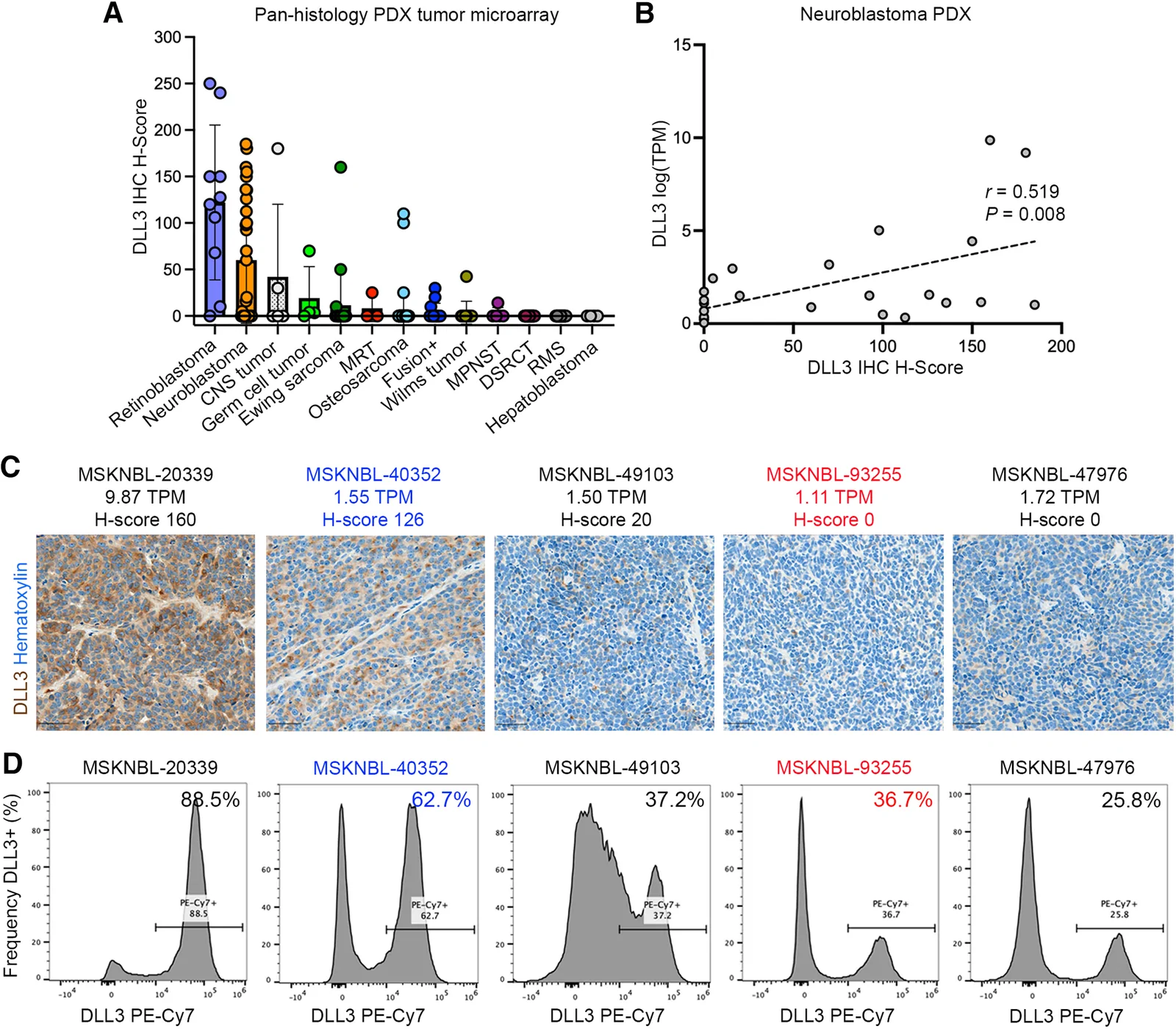
DLL3 expression and cell surface localization in preclinical neuroblastoma PDX tumor models. (A) DLL3 H-scores across pan-histology PDX tumor microarray generated from 244 PDX models, including 30 neuroblastoma PDXs. (B) Correlation between DLL3 transcript levels determined by bulk RNA sequencing and DLL3 H-scores in 25 neuroblastoma PDX models. (C) Representative DLL3 IHC images from neuroblastoma PDX models with corresponding DLL3 transcript levels (TPM) and H-scores (scale bar, 50 µm). (D) Flow cytometric analysis of same PDX models stained with anti-DLL3 antibody. Histograms show DLL3-positive cells within CD45-negative, CD56-positive population using modal normalization. CNS = central nervous system; DSRCT = desmoplastic small round cell tumor; H-score = histochemical score; IHC = immunohistochemistry; MPNST = malignant peripheral nerve sheath tumor; MRT = malignant rhabdoid tumor; PE-Cy7 = phycoerythrin–cyanine 7; RMS = rhabdomyosarcoma.

Given the discordance between immunohistochemistry and cell surface DLL3 expression, we next sought to determine whether DLL3 is pharmacologically accessible in vivo. We performed PET imaging and biodistribution studies using a radiolabeled anti-DLL3 monoclonal antibody (SC16.56), the binder used in the DLL3-targeting antibody–drug conjugate rovalpituzumab tesirine. This antibody cross-reacts with human and murine DLL3, enabling in vivo evaluation of DLL3 surface expression in neuroblastoma PDX models and nonmalignant tissues. Imaging was performed in 2 previously characterized neuroblastoma PDX models that were DLL3-positive by flow cytometry (MSKNBL-40352, 63%; MSKNBL-93255, 37%), although only MSKNBL-40352 was DLL3-positive by immunohistochemistry (histochemical score, 160 vs. 0) (Figs. 3C and 3D).

DLL3 PET imaging demonstrated robust tumor uptake of this diagnostic tracer in both neuroblastoma PDX models, with the tumor-associated signal evident by 72 h and increasing over time (Fig. 4A). MSKNBL-40352 exhibited higher uptake than that of MSKNBL-93255 across all time points (MSKNBL-40352 at 24 h, 7.21 %ID/g; 72 h, 13.11 %ID/g; 120 h, 21.63 %ID/g; MSKNBL-93255 at 24 h, 4.20 %ID/g; 72 h, 6.73 %ID/g; 120 h, 8.67 %ID/g) (Fig. 4A).

**FIGURE 4.**
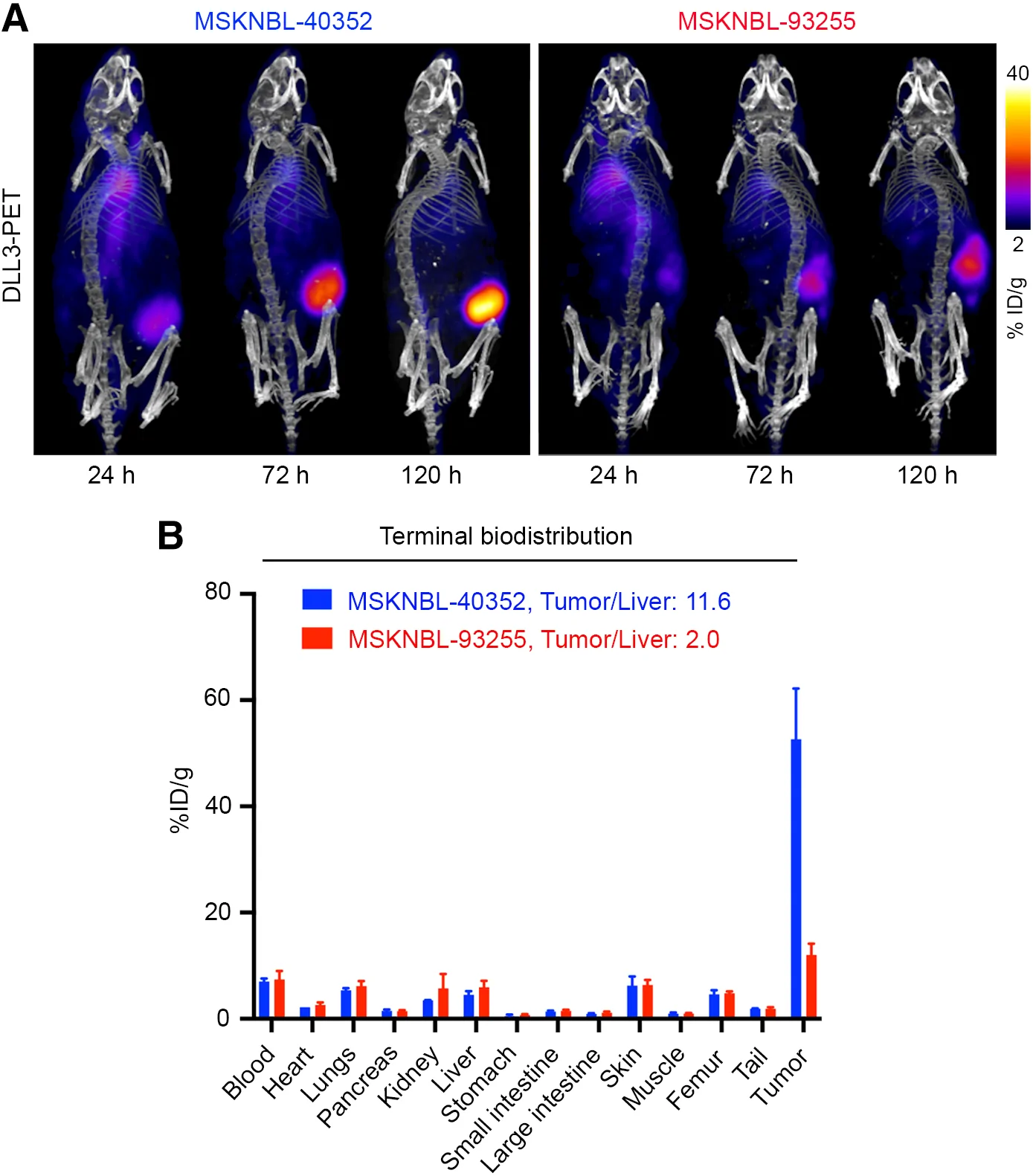
DLL3 PET imaging in neuroblastoma PDX models. (A) In vivo DLL3 PET imaging of PDX models after injection of [^89^Zr]Zr-deferoxamine-SC16.56. Maximum intensity projections of tumor uptake (%ID/g) at 24, 72, and 120 h were overlaid on CT images. (B) Terminal biodistribution of DLL3 PET radiotracer uptake in tumor and normal tissue from MSKNBL-40352 cohort (*n* = 2) and MSKNBL-93255 cohort (*n* = 4), plotted as %ID/g.

Biodistribution analysis at 120 h confirmed tumor accumulation in both models, with greater uptake in the higher DLL3–expressing MSKNBL-40352 tumors (52.65 %ID/g) than in the MSKNBL-93255 tumors (12.03 %ID/g) (Fig. 4B). Minimal tracer uptake was observed in blood (7.02 %ID/g) and normal tissues, including muscle (0.96 %ID/g), kidney (3.48 %ID/g), and liver (4.54 %ID/g), consistent with the negligible DLL3 expression previously documented in nonmalignant tissues (Fig. 4B) (2–4,13). Tumor-to-liver ratios of 11.6 for MSKNBL-40352 and 2.0 for MSKNBL-93255 support tumor-specific uptake.

### DLL3 PET Imaging in Patients with Neuroblastoma

We performed [^89^Zr]Zr-deferoxamine-SC16.56 PET imaging in 4 young adults (19–28 y) with relapsed, metastatic neuroblastoma (Table 1; Supplemental Table 1). These patients were enrolled as a predefined subgroup within a phase 2 DLL3 PET imaging trial of adult patients with neuroendocrine tumors. All patients presented with metastatic disease, received multiple prior lines of therapy, and had refractory disease. Two patients were diagnosed in childhood (age, 8 and 12 y), whereas the other 2 were young adults (age, 19 and 23 y) at the time of initial diagnosis. No patients had MYCN-amplified tumors (as expected for older patients), but patient 1 harbored *ATRX* and *ALK* gene mutations, which correlate with aggressive disease (Supplemental Table 1). No adverse events were observed.

**TABLE 1.** Patient Characteristics and DLL3 PET/CT Quantifications

Patient	Age at diagnosis (y)	Age at scan (y)	DLL3-avid lesions	Blood SUV_max_/ SUV_mean_	Liver SUV_max_/ SUV_mean_	Most DLL3-avid tumor	Tumor- to-liver ratio	Tumor- to-bone ratio
1	23	28	4	7.3/5.1	11.3/9.6	Left skull base (12)	1.3	10.9
2	12	19	3	10.3/8.5	8.4/5.6	Left ischial acetabulum (28.4)	5.1	23.7
3	8	22	>25	7.3/5.9	6.5/4.6	Right posterior iliac (34.5)	7.5	20.3
4	19	23	>20	8.2/4.8	15.8/12.0	Right anterior acetabulum (35.6)	3.0	32.4

Most DLL3-avid tumor is location with SUV_max_ in parentheses. Tumor-to-liver ratio and tumor-to-bone ratio are SUV_max_/SUV_mean_.

Tumor-specific uptake of [^89^Zr]Zr-deferoxamine-SC16.56 was detected in all 4 patients, irrespective of disease burden (patients 1 and 2, low burden; patients 3 and 4, high burden) (Table 1; Figs. 5 and 6). All lesions were identified on standard surveillance imaging, including [^123^I]MIBG scans, and exhibited concordant DLL3 PET avidity, with lesion SUV_max_ ranging from 12 to 35.6. Symmetric physiologic uptake in the parotid and submandibular glands, known sites of MIBG accumulation, was excluded from disease assessment (Figs. 5A and 5B).

**FIGURE 5.**
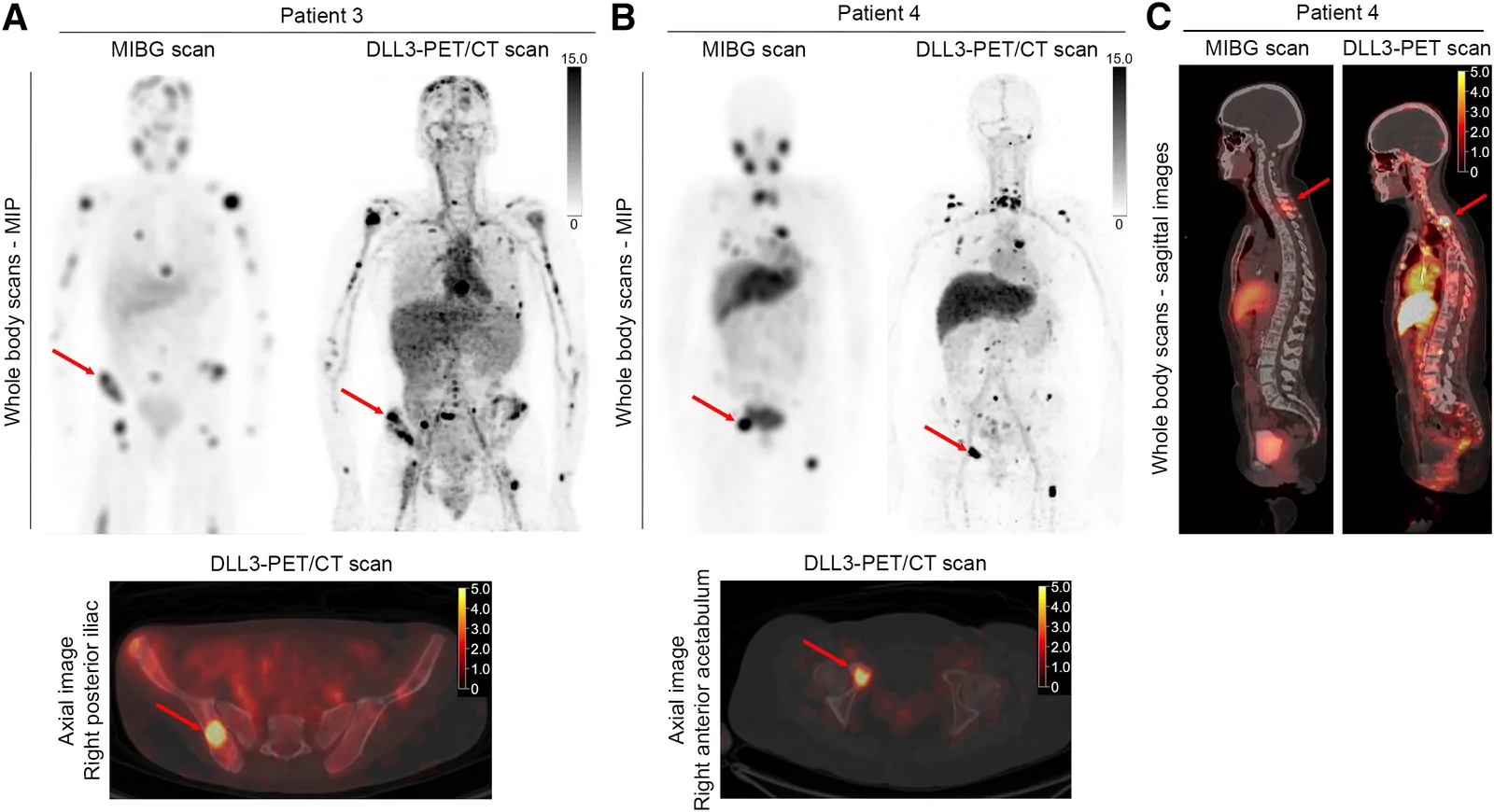
DLL3 PET imaging in patients with widespread refractory neuroblastoma. (A–C) [^89^Zr]Zr-deferoxamine-SC16.56 PET/CT imaging from patients 3 and 4. MIP MIBG and DLL3 PET/CT scans from patient 3 (A) and patient 4 (B and C) demonstrate concordant uptake in metastatic lesions, with corresponding axial images below. MIP = maximum-intensity projection.

**FIGURE 6.**
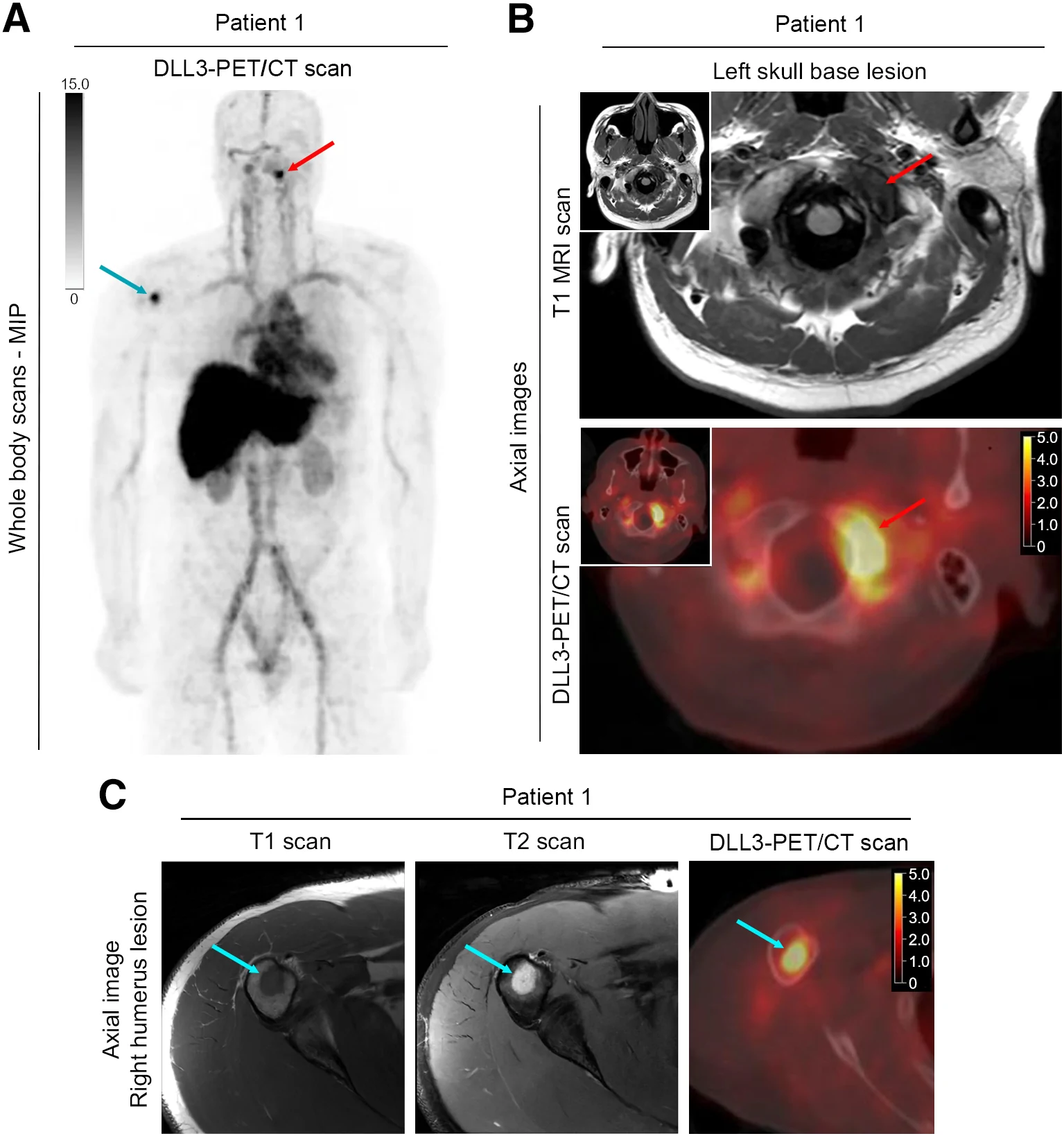
DLL3 PET imaging in patient with refractory neuroblastoma with low disease burden. (A–C) Whole-body DLL3 PET/CT MIP scan (A) and T1-/T2-weighted MRI scans from patient 1 highlight left skull base lesion (red arrow, B) and right humerus lesion (blue arrow, C). Axial images show T1-weighted scan for left skull base (B) and both T1- and T2-weighted scans for right humerus (C). MIP = maximum-intensity projection.

Representative DLL3 PET/CT images highlight the extent and intensity of DLL3 PET uptake across patients with variable disease burden. In patients with extensive metastatic disease (patients 3 and 4), DLL3 PET identified widespread osseous involvement. In patient 3, more than 25 bone lesions were visualized, and the right posterior iliac lesion had the highest uptake (SUV_max_, 34.5) (Fig. 5A). Similarly, in patient 4, more than 20 bone lesions were detected, with particularly avid uptake in lesions involving the right acetabulum and thoracic spine (Figs. 5B and 5C). An antecedent biopsy of a left paraspinal mass was DLL3-positive by immunohistochemistry (Supplemental Fig. 3A). DLL3 PET also detected all known lesions in 2 patients with low disease burden: a left skull base and right humeral lesion in patient 1, both confirmed on dedicated MRI (Fig. 6), and 3 osseous lesions in patient 2, including a left ischial-acetabular lesion (SUV_max_, 28.4) (as shown in the graphical abstract).

Lesion-specific uptake was quantified using tumor-to-bone and tumor-to-liver ratios, which ranged from 10.9 to 32.4 and 1.25 to 7.52, respectively (Table 1). These results demonstrate in vivo targeting of [^89^Zr]Zr-deferoxamine-SC16.56 to DLL3-expressing tumor sites and establish the accessibility of DLL3 in patients with neuroblastoma.

### DLL3 Cell Surface Expression Is Independent of MYCN Status and Poorly Predicted by Immunohistochemistry

Because of age restrictions, our initial DLL3 PET/CT cohort consisted exclusively of adolescent and young adult patients with MYCN-nonamplified neuroblastoma. To determine whether DLL3 surface expression correlated with MYCN-amplified disease, we analyzed all preclinical samples (*n* = 11) and primary patient samples (*n* = 7) evaluated by flow cytometry (*n* = 18 total), including 8 MYCN-nonamplified samples and 10 MYCN-amplified samples. MYCN status was not associated with a statistically significant difference in DLL3 cell surface expression, with a trend toward a higher fraction of DLL3-positive cells in MYCN-amplified samples (Fig. 7A).

**FIGURE 7.**
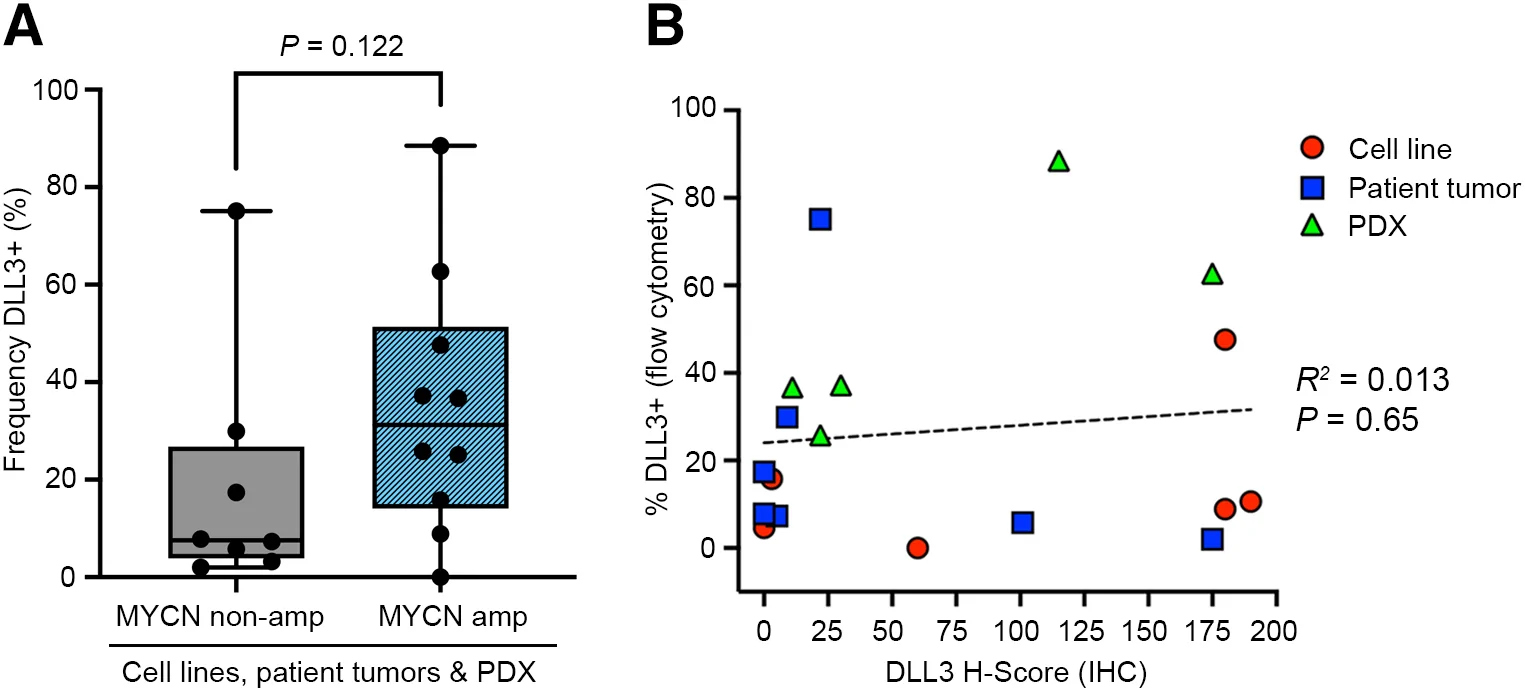
Relationship among DLL3 cell surface expression, MYCN status, and DLL3 immunohistochemistry. (A) Association between MYCN amplification and percentage of DLL3-positive tumor cells measured by flow cytometry across neuroblastoma cell lines, PDX models, and patient tumors (Mann–Whitney test). (B) No correlation between DLL3 H-scores and DLL3-positive tumor cells measured by flow cytometry across patient and preclinical models (*n* = 18). amp = amplified; H-score = histochemical score; IHC = immunohistochemistry.

To determine whether DLL3 immunohistochemistry could serve as a biomarker for selecting patients for DLL3-targeted therapies, we assessed the relationship between DLL3 immunohistochemistry staining and cell surface DLL3 expression measured by flow cytometry. After aggregation of data across all available neuroblastoma sample types (7 patient samples, 5 PDX tumors, and 6 cell lines), there was no correlation between DLL3 histochemical score and cell surface DLL3 expression (Fig. 7B). These findings indicate that DLL3 immunohistochemistry does not reliably predict cell surface DLL3 expression.

## DISCUSSION

DLL3 is a well-established therapeutic target in adult neuroendocrine tumors, and we provide clinical and translational evidence supporting DLL3 as a biologically actionable target in neuroblastoma, a pediatric tumor of neuroendocrine derivation. Across all neuroblastoma samples analyzed, including patient tumors, PDXs, and cell lines, most demonstrated cell surface DLL3 expression, including tumors classified as DLL3-negative by immunohistochemistry. In aggregate, 18 primary neuroblastoma tumor samples, neuroblastoma PDX tumors, and neuroblastoma cell lines were assessed using flow cytometry, and a single sample (KELLY cell line) demonstrated an absence of DLL3 at the cell surface. DLL3 abundance based on median fluorescence intensity in neuroblastoma cells also appeared comparable to that observed in small cell lung cancer (Supplemental Fig. 2A). Because several therapeutic modalities, including antibody–drug conjugates, radioligands, bispecific T-cell engagers, and chimeric antigen receptor T cells, exert antitumor activity even with modest target expression (21), DLL3 surface expression may permit targeting in a broader cohort of patients with neuroblastoma than previously appreciated by immunohistochemistry.

Although DLL3 protein levels assessed by immunohistochemistry were generally correlated with transcript levels, histochemical score, calculated from staining intensity and fraction, did not predict cell surface expression and localization, highlighting an important limitation of conventional tissue-based assays for the identification of therapeutically targetable DLL3. This discordance likely reflects that under normal physiologic conditions, DLL3 localizes predominantly in the Golgi apparatus (11,14,22). In neuroendocrine malignancies, DLL3 is transcriptionally upregulated, and its overexpression may contribute to tumor growth and survival, supporting its role as a biologically relevant dependency (12,13,15). Despite overexpression, DLL3 remains predominantly intracellular, consistent with its role in Notch pathway regulation (23). Although the mechanisms governing trafficking remain incompletely defined, likely involving dysregulated protein processing and intracellular trafficking, pathologic overexpression is associated with detectable cell surface localization. This redistribution may be difficult to discern by immunohistochemistry, particularly in cases with heterogeneous expression. In addition, discordance may be due to fixation-dependent epitope masking and restricted antigen accessibility in immunohistochemistry, which disproportionately affect detection of low-level or heterogeneous protein expression (24,25). These findings suggest immunohistochemistry may not reliably identify patients with therapeutically targetable DLL3 and support the development of alternative approaches to identify tumors with surface-accessible DLL3 for clinical trials.

Although flow cytometry provides direct confirmation of DLL3 surface localization, its limited availability and requirement for fresh tissue limits its feasibility as a biomarker for patient selection. DLL3-targeting PET imaging offers a compelling solution to identify DLL3-positive disease and patients who may benefit from DLL3-targeting therapeutics. Our PDX studies, using a DLL3 probe with cross-species specificity, demonstrated selective tumor uptake with minimal background signal, even in tumors with low DLL3 expression. Although PDX models lack the native human tumor microenvironment and the mouse stroma can influence protein processing, trafficking, or cell surface localization, concordant DLL3 surface expression in primary patient tumors by flow cytometry supports that DLL3 surface localization is largely tumor-intrinsic and not dependent on stromal context. Consistent with this, DLL3 PET in all 4 patients with neuroblastoma demonstrated tumor-specific uptake, detecting all lesions identified on standard surveillance imaging. Although we acknowledge the small cohort size, these consistent findings provide early clinical evidence that DLL3 is accessible to antibody-based targeting.

Although our initial human imaging cohort did not include patients with MYCN-amplified disease, MYCN amplification will be prevalent among patients enrolling in any early-phase clinical trial of DLL3-targeted therapeutics. DLL3 surface expression was preserved in MYCN-amplified tumors. All 5 MYCN-amplified PDX models assessed by flow cytometry demonstrated detectable DLL3 cell surface expression, and MYCN-amplified tumors tended to harbor a higher fraction of tumor cells with surface-accessible DLL3. In addition, both PDX models evaluated using DLL3 PET imaging were MYCN-amplified. Together, these findings suggest that DLL3-directed therapies may be applicable across molecular subtypes of neuroblastoma, including MYCN-amplified disease.

The DLL3-specific PET imaging results in patients with neuroblastoma rationalize a theranostic approach in which a therapeutic radioligand (e.g., ^177^Lu) is applied to patients with target-avid disease determined by PET imaging, analogous to the theranostic approach to PSMA-expressing prostate cancer (26). Given the heterogeneous expression of DLL3 within tumors, therapeutic payloads with a greater field effect (e.g., immune cell targeting or radioligands) may be particularly well suited for patients with DLL3-expressing neuroblastoma. Food and Drug Administration approval of the DLL3-bispecific T-cell engager, tarlatamab, demonstrates that DLL3-directed immune engagement can be both effective and well tolerated (3,5,6). Preclinical and clinical data suggest that tarlatamab retains activity in tumors with low DLL3 expression or DLL3 undetectable by immunohistochemistry, underscoring the importance of identifying patients with modest but targetable surface expression (5,6,27). In the DeLLphi-301 phase 1 trial, objective responses were observed irrespective of DLL3 status as shown by immunohistochemistry, and DLL3 testing was not required for enrollment in the subsequent phase 3 study (5,6,28). Similarly, because a substantial proportion of neuroblastoma samples have demonstrable cell surface expression of DLL3, initial clinical testing of DLL3-targeted therapies in neuroblastoma should not be biomarker-restricted. Correlative assessment of clinically applicable measures of DLL3 in such a study will be required to identify an optimum biomarker strategy that predicts response, as was done for neuroendocrine prostate cancer (29).

## CONCLUSION

Survival for patients with high-risk neuroblastoma is less than 50% (9,10), underscoring the need for additional therapeutic approaches for neuroblastoma. This work provides a rationale for the clinical evaluation of DLL3-targeted therapies in neuroblastoma and supports the development of DLL3-based theranostic approaches for this disease.

## DISCLOSURE

This work was supported in part by the NIH (U01CA263967 to Andrew Kung and Filemon Dela Cruz, R01CA213448 to John Poirier, R35 CA263816 to Charles Rudin, and R35 CA232130 to Jason Lewis), the RBC Initiative in Immuno-Oncology, a Prostate Cancer Foundation TACTICAL Award (to Jason Lewis), the Druckenmiller Foundation, NIH Cancer Center support grant P30 CA008748, a National Center for Advancing Translational Sciences grant (UL1TR002384) of the Clinical and Translational Science Center at Weill Cornell Medical College, and Cookies for Kids’ Cancer (to Jason Lewis). No other potential conflict of interest relevant to this article was reported.

## Notes

KEY POINTS **QUESTION:** Is DLL3 expressed on the cell surface and accessible for in vivo targeting in patients with neuroblastoma? **PERTINENT FINDINGS:** In a translational study that integrated multiple modalities across preclinical models, human neuroblastoma tumor samples, and 4 patients with relapsed disease, flow cytometry demonstrated DLL3 cell surface expression across tumor samples, including those with low or absent immunohistochemistry expression. ^89^Zr-labeled DLL3 PET imaging confirmed in vivo tumor accessibility, with tumor-specific uptake observed in all patients and detection of all lesions identified on standard surveillance imaging. **IMPLICATIONS FOR PATIENT CARE:** DLL3 represents a clinically accessible therapeutic target in neuroblastoma, and DLL3-directed PET imaging may enable noninvasive identification of patients suitable for DLL3-targeted therapies.
